# Cultural variation in trust and acceptability of artificial intelligence diagnostics for dementia

**DOI:** 10.1177/13872877251319353

**Published:** 2025-02-16

**Authors:** Avinash Chandra, Kaviya Senthilvel, Rifah Anjum, Ijeoma Uchegbu, Laura J Smith, Helen Beaumont, Reshma Punjabi, Samina Begum, Charles R Marshall

**Affiliations:** 1 Centre for Preventive Neurology, Wolfson Institute of Population Health, Queen Mary University of London, London, UK; 2 School of Biological and Behavioural Sciences, Queen Mary University of London, London, UK; 3 AINOSTICS LTD, 3 Hardman Square, Spinningfields, Manchester, UK; 4 ABATED Study Lay Steering Committee Member, UK

**Keywords:** acceptability, Alzheimer’s disease, artificial intelligence, dementia diagnosis, diagnostics, health inequalities, machine learning, trust

## Abstract

Digital health innovations hold diagnostic and therapeutic promise but may be subject to biases for underrepresented groups. We explored perceptions of using artificial intelligence (AI) diagnostics for dementia through a focus group as part of the Automated Brain Image Analysis for Timely and Equitable Dementia Diagnosis (ABATED) study. Qualitative feedback from a diverse public engagement group indicated that cultural variations in trust and acceptability of AI diagnostics may be an unrecognised source of real-world inequity. Efforts focused on the adoption of AI diagnostics in memory clinic pathways should aim to recognise and account for this issue.

Digital health innovations have the potential to both mitigate or widen existing health inequalities in access to timely and accurate dementia diagnosis. Whereas improved diagnostic accuracy might ensure more uniform access to gold-standard care, biases introduced in the development of digital technology might disadvantage underrepresented demographic groups. This has been shown in other disease areas through the discovery of harmful racial variation in blood oxygen saturation measurements from pulse oximetry.^1^ Efforts to improve demographic representation in the development of medical artificial intelligence (AI) technologies should help to eliminate this bias. A risk is that new technologies may introduce inequity in real-world use due to cultural variation in perceptions of trust and acceptability. This aspect is less apparent during research and development and requires dedicated efforts including consultation with community stakeholders and the public.

We explored this issue through the Automated Brain Image Analysis for Timely and Equitable Dementia Diagnosis (ABATED) study.^2^ AI algorithms are increasingly showing promise in improving access to a dementia diagnosis,^3^ particularly for those with early cognitive symptoms who might benefit most from emerging treatments but often have to wait years to determine whether their condition has a neurodegenerative basis.^4^ This delay may be worse for ethnically diverse and socially deprived individuals, who are at greater risk of dementia, and are frequently underdiagnosed.^5,6^ For example, individuals in East London face delays in diagnosis due to cultural factors.^7^ The ABATED study aims to evaluate how well AI technology for dementia diagnosis translates into a real-world cohort of patients from memory clinics in East London, one of the most diverse and deprived populations in England.

In November 2023, we hosted a public engagement activity (focus group) as part of our study. Fifteen community members attended, including representatives of Black and South Asian community groups in East London and with lived experience of dementia. Focus group members were recruited through direct outreach to local community groups, the NIHR people in research platform, and from the ABATED steering committee. A semi-structured topic guide was designed to capture perceptions of diagnostic software, preferences around information received, and how this should be communicated to patients. Members of the ABATED study team facilitated and contributed to discussions. The focus group was audio-recorded and field notes taken. Upon completion, the research team reviewed raw data. A qualitative approach was taken to systematically identify and label specific responses using codes. These codes were then collated, reviewed, and refined by the research team to extract key themes to explain meaning across the dataset. Two key themes were identified in relation to the equitable implementation of medical diagnostic innovations in memory clinic pathways

The first was that perceived trustworthiness varied depending on the terms used. Participants welcomed diagnostic software innovation, but many expressed discomfort when the technology was described as “artificial intelligence” or “AI”, preferring descriptions such as “computer software” or “developed using machine learning”. Negative connotations about AI tended to relate to fears about computer systems demonstrating excess agency and generalising learning to new domains and tasks. One participant acknowledged that a change in terminology may be construed as disingenuous, further reducing trust. Use of an algorithm trained on data modalities for a specific purpose was seen as sufficiently distinct from the group’s conceptions of AI that, overall, they felt the alternative descriptors were more useful and informative. This is consistent with recent evidence that mistrust in AI systems may not be wholly grounded in issues of operability or influence of AI, but internal biases and beliefs.^8^

The second emerging theme was that the group unanimously placed more trust in clinicians than in medical software and therefore preferred to be told that a clinician had made a dementia diagnosis that factored in information from a computerised diagnostic decision aid, and not that the diagnosis had been made by a computer. The group expressed concerns about a potential lack of training on how to effectively use AI, especially for newer health care professionals, who they worried might exhibit an over-reliance on AI technology to generate diagnostic reports at the expense of exercising clinical judgement. This aligns with recommendations that AI should serve an assistive role within the healthcare system, and training be incorporated in medical curricula.^9^ Other suggestions from the group were that healthcare professionals should (1) emphasise that AI is one of the many tools in the clinician’s toolbox, and (2) acknowledge the predictive accuracy for the AI and (3) identify any potential biases, such as those based in ethnicity.^8^ This latter point may entail being upfront about the frequency of diagnostic errors in medical practice,^10^ and how AI, as a more objective measure, may be one way to reduce their occurrence.

The widespread adoption of AI technology has the potential to transform the timeliness and accuracy of a dementia diagnosis. However, this is only likely to mitigate health inequities if issues of trust and acceptability are addressed across diverse cultural backgrounds. Discussions from our focus group indicated that these might include careful selection of nomenclature for technology informed by AI, and explicitly maintaining the primacy of clinician judgement whilst incorporating digital decision aids. Future work should aim to validate the preliminary insights gained from this public engagement activity using quantitative approaches (e.g., structured survey results), in larger samples, and across a range of different cultural groups.
